# Renin-Angiotensin System Blockers May Prolong Survival of Metastatic Non-Small Cell Lung Cancer Patients Receiving Erlotinib

**DOI:** 10.1097/MD.0000000000000887

**Published:** 2015-06-05

**Authors:** Adnan Aydiner, Rumeysa Ciftci, Fatma Sen

**Affiliations:** From the Department of Medical Oncology, Istanbul University, Institute of Oncology, Capa, Istanbul, Turkey (AA, RC, FS).

## Abstract

The aim of this study is to determine whether renin-angiotensin system blockers (RASBs), which include angiotensin-converting enzyme inhibitors (ACEIs) and angiotensin-2 receptor 1 blockers (ARBs), improve the overall survival (OS) of patients with metastatic non-small cell lung cancer (NSCLC).

The medical charts of 117 patients with metastatic NSCLC were retrospectively assessed. Thirty-seven patients (RASB group) using RASBs during systemic treatment were compared with 80 controls (control group) who did not use RASBs following the diagnosis of NSCLC. The histological tumor subtype, performance status, age, sex, smoking status, comorbidities, other medications, chemotherapeutics (CT), and erlotinib that were received in any line of treatment were recorded. We compared the OS of the patients in the RASB and control groups.

The median (±SD) age of the patients was 61 (±1) years and all patients were administered systemic treatment (CT or erlotinib). The patients in RASB group were more likely to be smokers, have hypertension and ischemic heart disease, and use erlotinib, thiazides, beta-blockers, and calcium-channel blockers (*P* < 0.05 for all) compared with the control group. The median follow-up time was 18.9 months (range 1–102 months) for the entire group. The median follow-up period was longer for RASB group than control group (17 vs 11 months, *P* = 0.033). The most commonly prescribed RASB agent was valsartan (n = 12/37). At the time of the analysis, 98 (83.7%) of all patients had died. In the univariate analysis, the median OS was longer in the RASB group compared with the control group (17 [±4.1] vs 12 [±1.4] months, *P* = 0.016). Interestingly, further analyses revealed that RASBs significantly improved OS only if used with erlotinib concurrently (34 [±13.8] vs 25 [±5] months, *P* = 0.002) and the OS benefit was more attributable to ARBs because only 4 patients received ACEI and erlotinib concurrently. However, the benefit of ARBs on OS disappeared in the multivariate analysis.

The use of ARBs during erlotinib treatment may prolong OS of patients with metastatic NSCLC.

## INTRODUCTION

Lung cancer is a common cause of cancer death worldwide, despite all of the current cancer therapeutics.^1^ Eighty percent of lung cancer cases are non-small cell lung cancer (NSCLC), predominantly adenocarcinoma.

The renin-angiotensin system (RAS) is mainly associated with the regulation of arterial pressure.^2–4^ RAS includes angiotensin (AT) 2, angiotensin-converting enzyme (ACE), AT (1–7), angiotensin receptors 1 and 2 (AT1R and AT2R), bradykinin and its receptors. AT2 has vasoconstrictor and mitogenic effects, whereas bradykinin has the opposite properties. AT2 also contributes to cell migration, inflammation, angiogenesis, and extracellular matrix formation via AT1R and AT2Rs.^2,4–6^ AT1Rs are responsible for the majority of well-known AT2 effects; however, the role of AT2R is less known. AT2Rs, which are mainly expressed on fetus and injured tissues, induce apoptosis.^4^

RASBs (renin-angiotensin system blockers) include ACE inhibitors (ACEIs) and AT1R blockers (ARBs) and have been commonly prescribed drugs, which have anti-hypertensive and end-organ protective properties.^4^ ARBs act on only AT1Rs, whereas ACEIs reduce production of AT2 and decrease activity of both AT1Rs and AT2Rs. ACEIs also cause accumulation of bradykinin and AT (1–7), which have anti-mitogenic, anti-angiogenic, and vasodilator effects.^2–4^

RAS components are expressed in a number of cancer types, such as carcinomas of the ovary, kidney, pancreas, stomach, and lung.^4,5,7–10^ Preclinical studies have demonstrated that RAS signaling has strong tumor-promoting effects in a number of cancer types, including NSCLC.^5,7,8,11^ In the past, it was shown that serum ACE level decreased and low levels had poor prognostic effect in NSCLC patients.^12^ AT1R signaling can transactivate some tyrosine kinases including epidermal growth factor receptor (EGFR), which stimulates pro-oncogenic pathways.^13^ Emerging data suggest that the tumoral local RAS system produces AT2, which induces epithelial-mesenchymal transition, tumor proliferation, and angiogenesis.^10,14^ Moreover, this effect could be reversed by RASBs,^5,8,11,15^ suggesting that these agents may be promising for the treatment of NSCLC.

Regarding the impact of RASBs on cancer survival, it has been shown that RASBs improve overall survival (OS) in some cancer types including NSCLC.^16–18^ Most of the preclinical studies investigating the role of RAS signaling on NSCLC carcinogenesis and progression are experimental.^5,7,8,11,14,19–21^ In a retrospective study, RASBs have been found to improve OS in advanced NSCLC when used with platinum-based chemotherapy (CT).^16^ However, patients receiving erlotinib or the other targeted therapies were not included in that study. In light of the available data, addition of RASBs to systemic anti-cancer therapy against advanced NSCLC may improve prognosis. Due to the lack of the clinical data that show whether RASBs have an impact on survival of patients with metastatic NSCLC, the present study was designed to compare the OS of patients with metastatic NSCLC according to the use of RASBs during CT or targeted therapy, retrospectively.

## PATIENTS AND METHODS

This case-control study was performed by retrospectively screening 850 NSCLC patients diagnosed between 2003 and 2011. We identified 37 patients with metastatic NSCLC receiving an RASB agent during CT or erlotinib treatment at any time after the diagnosis of the malignancy (RASB group). We evaluated whether the use of RASBs (as clinically indicated) had an effect on OS by comparing these 37 patients with 80 age-, sex-, and histological subtype-matched counterparts who did not use RASBs at any time after the diagnosis of NSCLC (control group). While selecting appropriate controls, we planned to match the RASB and control groups at a 1:2 ratio based on age, sex, ECOG performance status, stage, and histological subtype. The most appropriate matching control group included 6 more patients than the preplanned number. This study is a retrospective review of 117 patients with metastatic NSCLC who were diagnosed between 2003 and 2011. All of the patients enrolled in the study had pathologically confirmed NSCLC, distant metastasis, and an ECOG performance status of 0 to 3 and received CT and/or erlotinib, if a sensitizing EGFR mutation was detected. Patients with a history of another malignancy, who could not receive any systemic anti-cancer treatment or with an ECOG performance status of 4, were excluded.

Data about the medications of the patients were recorded from their medical charts. Patients were included in the RASB group if they used an ARB or ACEI agent during CT or erlotinib treatment at any time after the diagnosis of NSCLC, and patients who did not receive an ARB or ACEI agent at any time after their cancer diagnosis were included in the control group. The parameters that may affect the outcome of NSCLC and thus RASB analysis, such as histological tumor subtype, performance status (ECOG), age, sex, smoking status, comorbidities (hypertension [HT], ischemic heart disease (IHD), and diabetes mellitus DM]) and other medications, including anti-aggregants, thiazides, beta-blockers (BBs), calcium channel blockers (CCBs), chemotherapeutics, and erlotinib (an EGFR tyrosine kinase inhibitor) that were received in any line of treatment were recorded.

We analyzed the OS, which was defined as the time elapsed from the date of diagnosis to the date of death from any cause. For OS, death was an event time. The follow-up time was defined as the time from the date of diagnosis to the date of death or last follow-up.

The statistical analyses were conducted using Statistical Package for Social Sciences (SPSS) version 16 (SPSS Inc, Chicago, IL). The categorical variables were compared using Pearson *χ*^2^ test in the RASB and control groups. Student *t* test and Mann–Whitney *U* test were used to compare the normally and non-normally distributing numerical variables between groups, respectively. Univariate analysis was performed by using the Kaplan–Meier method to estimate the OS of different patient groups, and the groups were compared with the log-rank test. Cox regression analysis was used to determine the association of ARB usage with the OS in the multivariate analysis. In the multivariate analysis, confounders were included if they were significant at a 0.05 level in the univariate analysis (log rank test) or thought to be important for OS or the ARB effect. The proportional hazards assumption and model fit was assessed by means of residual (Schoenfeld and Martingale) analysis. The results were expressed as median OS ± SE (standard error) and hazard ratios (HRs) with 95% confidence intervals (CIs). A *P* value of <0.05 was considered statistically significant.

Ethical approval was not required for this retrospective study, as it did not relate to patient's privacy or treatment options. The informed consent for systemic anti-cancer therapies was obtained from all of the patients. However, we could not obtain a specific informed consent for this retrospective analysis, because most of the patients were dead or could not be contacted at the time of analysis.

## RESULTS

The current study included 117 patients with metastatic NSCLC. The median (±SD) age of the patients was 61(±1) years. RASB and control groups included 37 (31.6%) and 80 (68.4%) patients, respectively. Most of the patients were male, smokers and more likely to have non-squamous histology and good performance status. Patients in the RASB group were more likely to have HT and IHD, as expected. The proportions of patients using thiazides, BBs, CCBs, and erlotinib were significantly larger in the RASB group than the control group. The median follow-up time was 18.9 months (range 1–102 months) for the entire group. The median follow-up period was longer for the RASB group than the control group (17 vs 11 months, p = 0.033). The patient characteristics and comparison of RASB and control groups are summarized in Table 1. In the RASB group, ARBs were primarily used (n = 21/37) and valsartan was the most commonly prescribed agent (n = 12/37). The prescribed ARBs were valsartan (12/21), losartan (3/21), candesartan (2/21), irbesartan (2/21), and olmesartan (2/21). The ACEIs used in our study were ramipril (5/16), trandolapril (4/16), lisinopril (3/16), cilazapril (2/16), fosinopril (1/16), and perindopril (1/16). At the time of the analysis, 98 (83.7%) of all patients (82.5% of the control group and 86.5% of the RASB group) had died. We also re-classified patients according to erlotinib, ACEI, and ARB usage (Tables 2 and 3). Seventeen of the 28 erlotinib users were RASB users simultaneously. Among the erlotinib users, the number of ARB and ACEI users was 13 and 4, respectively.

**TABLE 1:**
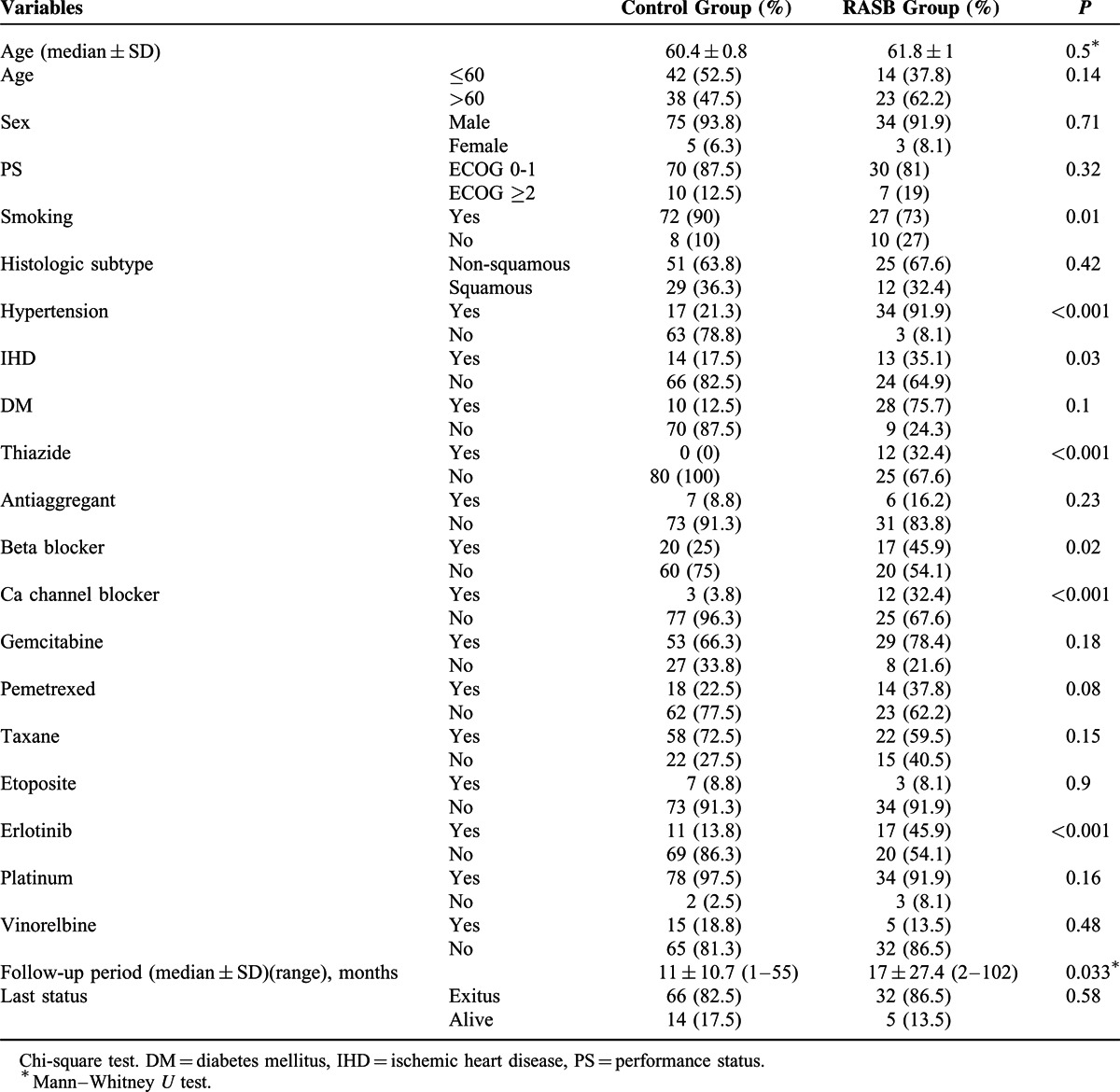
Patient Characteristics and Comparison of RASB and Control Groups

**TABLE 2:**
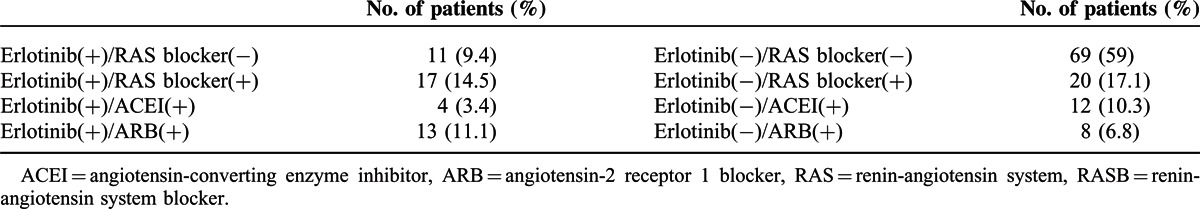
Patient Subgroups According to Erlotinib and RASB Usage

**TABLE 3:**
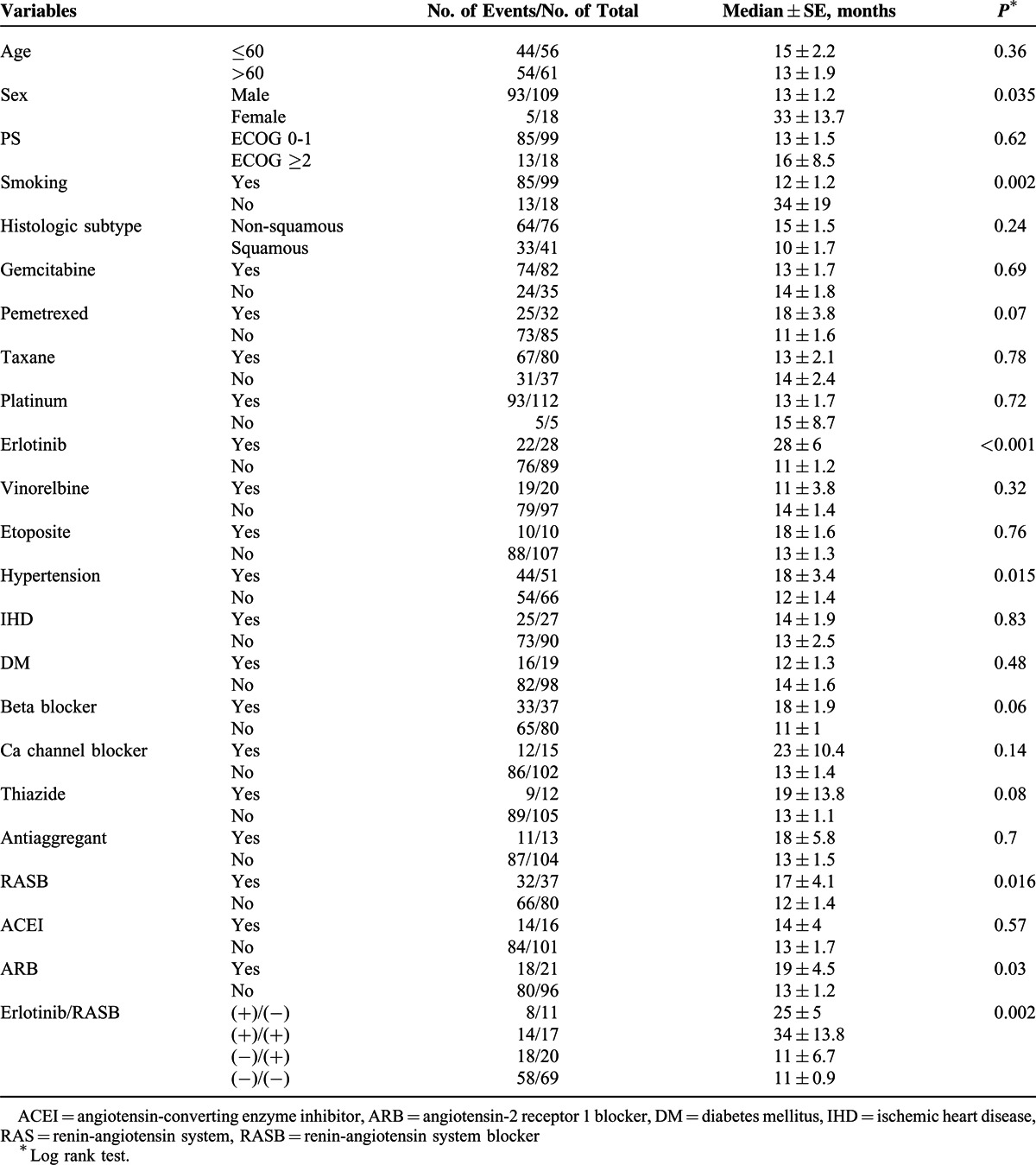
Univariate Analysis of OS

**FIGURE 1:**
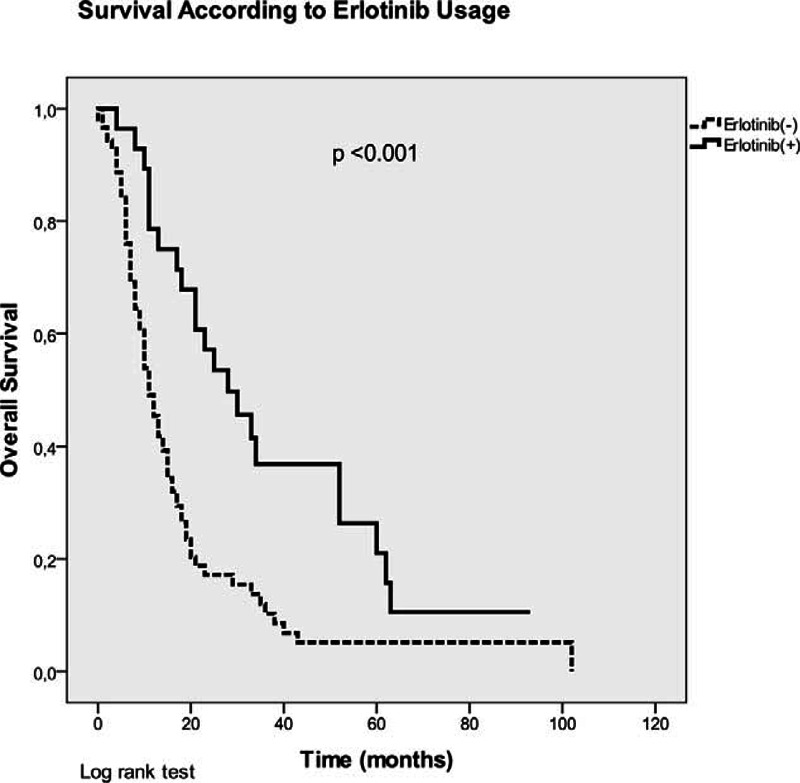
Kaplan–Meier curves of patients (overall survival) according to erlotinib usage.

**FIGURE 2:**
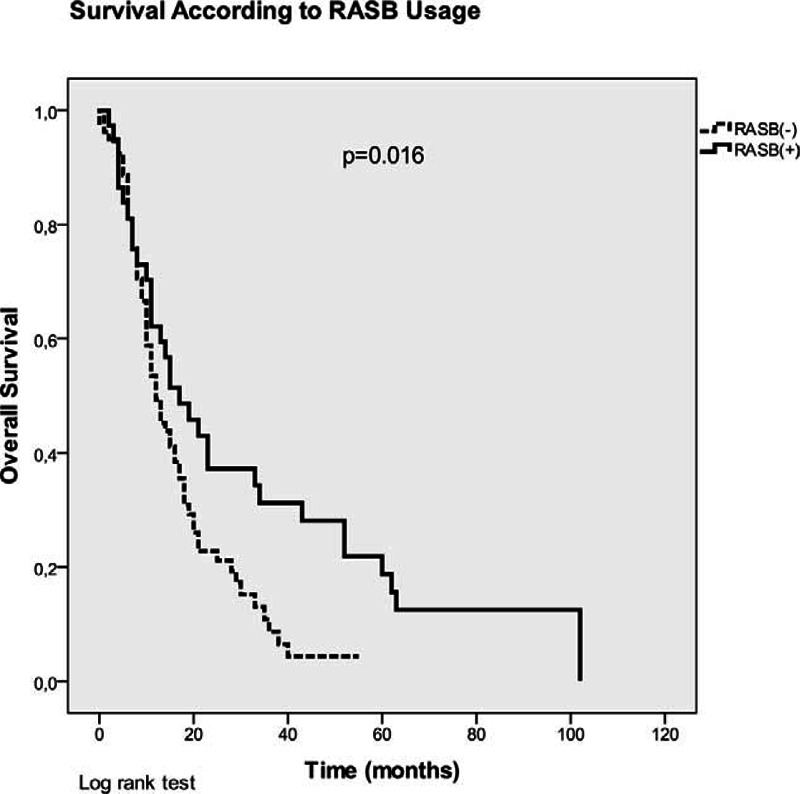
Kaplan–Meier curves of patients (overall survival) according to RASB usage.

The median OS of the entire group was 13 (±1.5) months. Female sex (33 vs 13 months, *P* = 0.035), using erlotinib (28 vs 11 months, *P* < 0.001), presence of HT (18 vs 12 months, *P* = 0.015), and using a RASB or ARB agent significantly improved OS in the univariate analysis, whereas smoking significantly decreased OS (Table 3, Figures 1 and 2). RASB and ARB usage provided 5-month (17 vs 12 months, *P* = 0.016) and 6-month (19 vs 13 months, *P* = 0.03) OS advantage, respectively (Figure 2). ACEI usage or the other variables examined in the univariate analysis revealed no significant impact on OS (Table 3).

**FIGURE 3:**
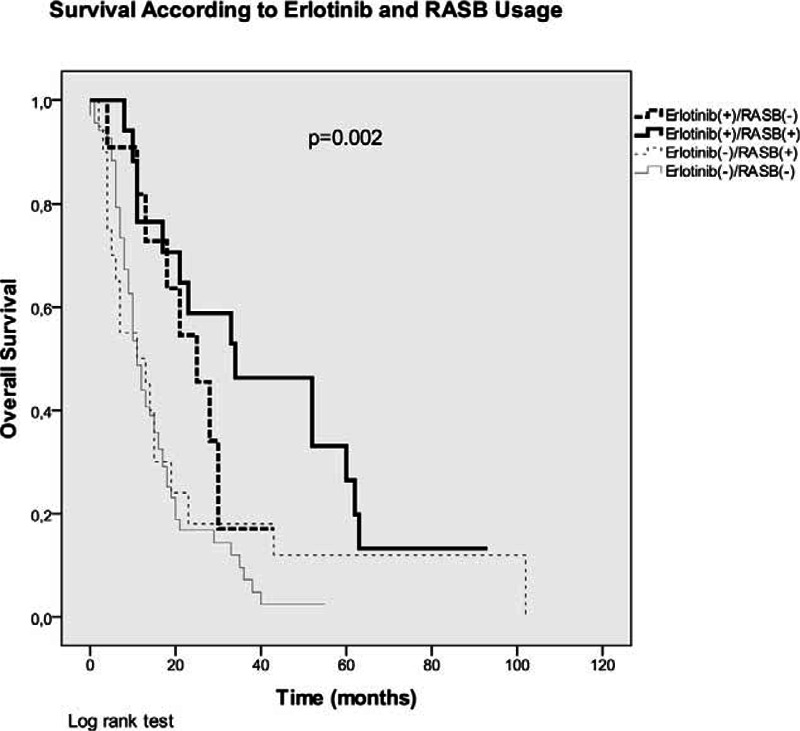
Kaplan–Meier curves of patients (overall survival) according to erlotinib and RASB usage.

After adjusting for age, sex, performance status, histological subtype, smoking status, presence of comorbidities (IHD, HT, and DM) and the use of erlotinib, the benefit of ARBs on OS disappeared in the multivariate analysis (HR: 0.99, 95% CI: 0.49–2, *P* = 0.98) (Table 4). Among these parameters, only erlotinib usage had a significant impact on OS (HR: 0.37, 95% CI: 0.17–0.76, *P* = 0.008). Therefore, we calculated the OS times of patients receiving an RASB drug according to erlotinib usage with a further analysis (Table 3). Erlotinib non-users had significantly worse OS (11 months) than erlotinib users (28 months) and RASB usage did not have an impact on OS among erlotinib non-users (Figure 3). However, RASB usage revealed a significant OS advantage (34 vs 25 months, *P* = 0.002) among erlotinib users (Figure 3).

**TABLE 4:**
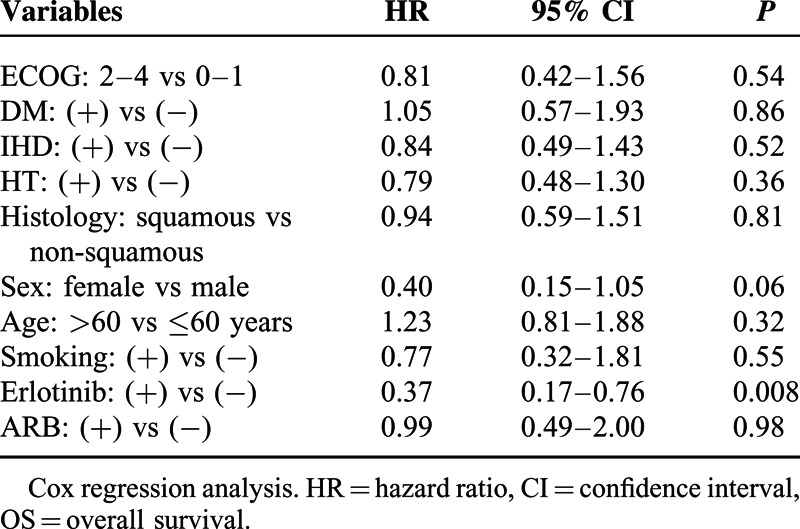
Multivariate Analysis of OS

## **D**ISCUSSION

This retrospective analysis showed that addition of RASBs, particularly ARBs, to systemic anti-cancer therapy was associated with better survival in metastatic NSCLC. In fact, the survival benefit of ARBs was dependent on erlotinib usage. However, survival advantage of ARBs disappeared in the multivariate analysis. The best outcomes were observed among erlotinib users receiving ARBs additionally. This may partly explain why the benefit of ARBs disappeared in the multivariate analysis.

Recent studies have emphasized on the importance of RAS in the initiation and progression of cancer, affecting inflammation, angiogenesis, apoptosis, cell migration, and the epithelial-mesenchymal transition.^4,8,11^

AT1R signaling appears to be the main component of RAS involved in tumor growth by inducing vascular endothelial growth factor (VEGF) synthesis, angiogenesis, inflammation, upregulation of endothelial adhesion molecules and subsequent infiltration of inflammatory or cancer cells, trans-activation of multiple tyrosine kinases, and subsequent cell proliferation.^6,8^ AT1Rs^8,9^ and AT2Rs^9^ are over-expressed in cancer tissue and promote tumor progression.^9^ Both ARBs and ACEIs can reverse angiogenic and mitogenic effects of AT2 in cancer tissue.^8^

RAS contributes to lung carcinogenesis and tumor progression; however, the mechanism appears to be complex. ^4,10,14,16,21–24^ AT2 increases intracellular calcium in only lung cancer cell lines but not in healthy lung cells, and this effect could be reversed by losartan, an ARB.^20^ In fact, according to the preclinical studies, it is still not clear whether AT2 or its receptors promotes apoptosis or proliferation of lung cancer cells.^21–24^

ACE2, an enzyme that converts AT2 to vasodilator and anti-proliferative AT(1–7),^10,19^ has been demonstrated to prevent NSCLC metastasis by inhibiting epithelial-mesenchymal transition.^14^ Similarly, AT(1–7) inhibits NSCLC growth both in vitro and in vivo,^10,19^ suggesting that ACE2 has a crucial role in balancing vasoconstrictor/proliferative and vasodilator/anti-proliferative functions of the RAS system.^10^ ACEIs are not able to affect ACE2 function directly; however, ACEIs can upregulate ACE2 expression.^4^ In lung cancer tissue, ACE2 expression is lower, whereas AT2 concentration is higher compared with healthy tissue.^10^ Moreover, ACE2 over-expression can decrease VEGF and AT1R production.^10^ Our findings support these preclinical studies indicating the tumor-promoting effect of RAS and the anti-tumoral activity of RASBs in NSCLC.^8,10,14,19–24^

Neprilysin, another enzyme that degrades bradykinin and converts AT1 to AT(1–7), is expressed at high levels in the healthy lung and regulates bronchial smooth muscle tonus.^4^ Cigarette smoking, a documented risk factor for NSCLC, inactivates neprilysin in the lung. In addition, the enzyme level is lower in case of cigarette smoking and lung cancer.^25^

Another explanation for the association of RAS signaling with cancer growth may be the trans-activation of growth factor receptors such as EGFR.^13^ Stimulation of AT1R trans-activates intracellular carboxyl terminus of EGFR simultaneously, independently from EGFR ligands.^13^ In this regard, AT1R and EGFR share a mutual downstream signaling after AT2 stimulation. Therefore, it is not surprising that RASBs provided the best survival advantage when used concurrently with erlotinib, an EGFR-tyrosine kinase inhibitor, in our study.

Cancer patients frequently receive cardiovascular medications, including RASBs, because cardiovascular diseases are common in the population. RASBs may provide synergistic effects with systemic treatment of cancer by reducing AT2-mediated cell proliferation and angiogenesis as well as increasing the AT(1–7), which has the opposite effects. The impact of concurrent use of RASBs during lung cancer treatment on survival has not been well-established. RASBs can decompress tumoral vessels and improve tumoral perfusion and drug delivery as well as increasing efficacy of radiation therapy.^15,26^ Some studies showed that RASBs prolong survival when given with CT concurrently in some cancer types.^16–18^ Therefore, we aimed to assess whether the use of RASBs have an impact on OS of metastatic NSCLC patients. In the present study, the OS of patients with metastatic NSCLC was compared retrospectively according to the use of RASBs. Although the rates of serious comorbidities including HT and IHD were higher in the RASB group compared with the control group, RASB group had significantly longer OS. However, the survival benefit of RASBs and ARBs demonstrated in the univariate analysis disappeared in the multivariate analysis. Our RASB group was small-sized and the follow-up period was significantly shorter in the control group, which may explain why the OS benefit of the RASBs disappeared in the multivariate analysis.

There is only one study investigating the role of RASBs on survival of NSCLC.^16^ According to that study, concurrent use of RASBs with platinum-based CT provides a significant OS benefit in advanced NSCLC patients (n = 287).^16^ RASB users (n = 52) were compared with non-users retrospectively.^16^ The patients who were administered first-line platinum-based CT with a RASB drug had a 3-month OS advantage. The follow-up time was 8 months and 31% of the patients had altered renal function. Importantly, the study included ACEI users (n = 43) predominantly; thus, ARB users appear to be under-represented. In current practice, EGFR-targeted therapies are recommended at first-line treatment of NSCLC for appropriate patients. The patients receiving targeted therapies, such as erlotinib, were not included in this study and the duration of RASB usage was not clearly described. Our results are in accordance with this study in terms of the benefit of RASB usage in metastatic NSCLC. However, our patients had been prescribed ARBs predominantly and receiving an ACEI did not affect OS in our study. In addition, the usage of erlotinib was the only significant factor improving OS in both univariate and multivariate analysis. Because the rate of erlotinib users was significantly higher in the RASB group, we performed a further analysis and showed that the use of RASBs provides OS advantage in only erlotinib users. Minority of the erlotinib users (4/22) received ACEI, whereas majority of the ACEI users (12/16) did not receive erlotinib. Because the number of those using erlotinib and ACEI concurrently was inadequate (n = 4), the OS benefit is more attributable to ARBs among erlotinib users. Because the number of ACEI users was inadequate among erlotinib users and RASBs revealed OS benefit only if used with erlotinib, it is not surprising that ACEIs did not provide OS benefit in our study.

In our study, we focused on all-cause mortality rather than cancer-specific or cardiovascular mortality. We did not find any association between RASB usage and CT agents. The OS benefit of RASBs is most likely not related to CT agents or other clinicopathologic variables, apart from erlotinib. The use of RASBs, particularly ARBs, with erlotinib revealed the best survival outcomes. The mechanism may be the synergistic interaction of ARBs and erlotinib by the crosstalk between AT1Rs and EGFR or ARBs may delay erlotinib resistance hypothetically. To our knowledge, this study is the first showing that ARBs are associated with a better OS when used by metastatic NSCLC patients during erlotinib treatment.

Our study has some limitations. The study group was small but appropriately matched according to well-known prognostic factors. Because this study was retrospective, some data may be missing or incomplete, such as the timing, duration, or doses of the RASBs, but all data were obtained from the patients’ medical charts, not from the prescription database. Tumoral penetration or potency may be different among RASBs; thus, the type and formulation may be important for the OS benefit. In this study, the ARBs were primarily used. Valsartan and ramipril were the most commonly used ARB and ACEI, respectively. Therefore, the OS benefit may be attributable to ARBs, especially to valsartan, because there was an insufficient number of patients receiving the other ARB types or ACEIs. In this regard, stating that the OS benefit is valid for all RASB types and formulations is difficult. In addition, we do not know the duration of RASB usage before the systemic anti-cancer treatment or whether that factor is important. Our study does not answer the questions whether EGFR amplifications and type of mutations are important or the results are valid for all EGFR-targeting agents such as gefitinib. Despite these limitations, the results are encouraging for further prospective randomized studies.

## CONCLUSION

This analysis demonstrated that the use of RASBs, particularly ARBs (as clinically indicated), may significantly improve the OS of patients with metastatic NSCLC. The most OS benefit was observed when ARBs were used with erlotinib concurrently. ARBs may be an inexpensive, feasible, and effective method to prolong the survival of metastatic NSCLC patients when used concurrently during erlotinib treatment. The use of RASBs in combination with erlotinib and the other EGFR-targeting drugs is worthy of investigation. The prognostic effects of RASBs on metastatic NSCLC should be investigated in more comprehensive studies with larger datasets to evaluate the duration, timing, or type of RASBs and their influence on the survival.
